# Socioeconomic conditions and children's mental health and quality of life during the COVID-19 pandemic: An intersectional analysis

**DOI:** 10.1016/j.ssmph.2023.101472

**Published:** 2023-07-23

**Authors:** Elsa Lorthe, Viviane Richard, Roxane Dumont, Andrea Loizeau, Javier Perez-Saez, Hélène Baysson, Maria-Eugenia Zaballa, Julien Lamour, Nick Pullen, Stephanie Schrempft, Rémy P. Barbe, Klara M. Posfay-Barbe, Idris Guessous, Silvia Stringhini, for the SEROCoV-KIDS study group, Deborah Amrein, Isabelle Arm-Vernez, Andrew S. Azman, Antoine Bal, Michael Balavoine, Rémy P. Barbe, Hélène Baysson, Julie Berthelot, Patrick Bleich, Livia Boehm, Aminata R. Bouchet, Gaëlle Bryand, Viola Bucolli, Prune Collombet, Alain Cudet, Vladimir Davidovic, Carlos de Mestral, Paola D’Ippolito, Richard Dubos, Roxane Dumont, Isabella Eckerle, Nacira El Merjani, Marion Favier, Natalie Francioli, Clément Graindorge, Idris Guessous, Munire Hagose, Séverine Harnal, Samia Hurst, Laurent Kaiser, Omar Kherad, Julien Lamour, Pierre Lescuyer, Arnaud G. L’Huillier, Andrea Loizeau, Elsa Lorthe, Chantal Martinez, Stéphanie Mermet, Mayssam Nehme, Natacha Noël, Francesco Pennacchio, Javier Perez-Saez, Anne Perrin, Didier Pittet, Klara M. Posfay-Barbe, Jane Portier, Géraldine Poulain, Caroline Pugin, Nick Pullen, Viviane Richard, Frederic Rinaldi, Deborah Rochat, Cyril Sahyoun, Irine Sakvarelidze, Khadija Samir, Hugo Alejandro Santa Ramirez, Jessica Rizzo, Stephanie Schrempft, Claire Semaani, Silvia Stringhini, Stéphanie Testini, Yvain Tisserand, Deborah Urrutia Rivas, Charlotte Verolet, Jennifer Villers, Guillemette Violot, Nicolas Vuilleumier, Sabine Yerly, María-Eugenia Zaballa, Christina Zavlanou, Silvia Stringhini

**Affiliations:** a Unit of Population Epidemiology, Division of Primary Care Medicine, Geneva University Hospitals, Geneva, Switzerland; b Université Paris Cité, Inserm, INRAE, Centre for Research in Epidemiology and Statistics Paris (CRESS), Paris, France; c Department of Health and Community Medicine, Faculty of Medicine, University of Geneva, Geneva, Switzerland; d Department of Epidemiology, Johns Hopkins Bloomberg School of Public Health, Baltimore, USA; e Division of Child and Adolescent Psychiatry, Department of Woman, Child, and Adolescent Medicine, Geneva University Hospitals, Geneva, Switzerland; f Department of Woman, Child, and Adolescent Medicine, Geneva University Hospitals, Geneva, Switzerland; g Department of Pediatrics, Gynecology & Obstetrics, Faculty of Medicine, University of Geneva, Geneva, Switzerland; h Division of Primary Care Medicine, Geneva University Hospitals, Geneva, Switzerland; i University Center for General Medicine and Public Health, University of Lausanne, Lausanne, Switzerland

**Keywords:** Youth, Adolescents, Socioeconomic disparities, Health equity, MAIHDA

## Abstract

**Background:**

Children and adolescents are highly vulnerable to the impact of sustained stressors during developmentally sensitive times. We investigated how demographic characteristics intersect with socioeconomic dimensions to shape the social patterning of quality of life and mental health in children and adolescents, two years into the COVID-19 pandemic.

**Methods:**

We used data from the prospective SEROCoV-KIDS cohort study of children and adolescents living in Geneva (Switzerland, 2022). We conducted an intersectional Multilevel Analysis of Individual Heterogeneity and Discriminatory Accuracy by nesting participants within 48 social strata defined by intersecting sex, age, immigrant background, parental education and financial hardship in Bayesian multilevel logistic models for poor health-related quality of life (HRQoL, measured with PedsQL) and mental health difficulties (measured with the Strengths and Difficulties Questionnaire).

**Results:**

Among participants aged 2–17 years, 240/2096 (11.5%, 95%CI 10.1–12.9) had poor HRQoL and 105/2135 (4.9%, 95%CI 4.0–5.9) had mental health difficulties. The predicted proportion of poor HRQoL ranged from 3.4% for 6–11 years old Swiss girls with highly educated parents and no financial hardship to 34.6% for 12–17 years old non-Swiss girls with highly educated parents and financial hardship. Intersectional strata involving adolescents and financial hardship showed substantially worse HRQoL than their counterparts. Between-stratum variations in the predicted frequency of mental health difficulties were limited (range 4.4%–6.5%).

**Conclusions:**

We found considerable differences in adverse outcomes across social strata. Our results suggest that, post-pandemic, interventions to address social inequities in HRQoL should focus on specific intersectional strata involving adolescents and families experiencing financial hardship, while those aiming to improve mental health should target all children and adolescents.

## Highlights

• The interplay of social, economic, and demographic characteristics in shaping children's well-being is not well understood.
• Intersectional analysis identified heterogeneous and outcome-specific social patterns of HRQoL and mental health.
• Strata involving adolescents and families with financial hardship showed substantially worse HRQoL and parent-reported poor mental health.
• Between-stratum variations in the predicted frequency of mental health difficulties were limited.
• These results indicate which groups should be targeted by public health policies and interventions to address social inequities.

## Graphical abstract

**Figure:**
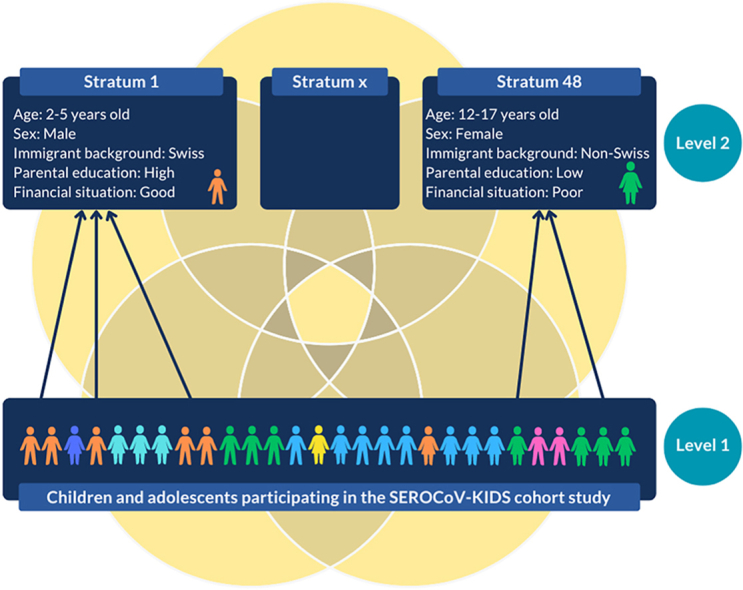

## Abbreviations

CrI: credible intervals
DA: Discriminatory Accuracy
HRQoL: Health-Related Quality of Life
MAIHDA: Multilevel Analysis of Individual Heterogeneity and Discriminatory Accuracy
MCMC: Markov chain Monte Carlo
MIPEX: Migrant Integration Policy Index
PCV: Proportional Change in Variance
PedsQL: Pediatric Quality of Life Inventory
SDQ: Strengths and Difficulties Questionnaire
VPC: Variance Partition Coefficient

## 1 Introduction

While the COVID-19 pandemic began as a health crisis, it quickly evolved into a full-blown economic and social crisis, the effects of which will probably cast a long shadow into the future. Children and adolescents faced massive disruptions to their daily lives and routines, prolonged periods of uncertainty, school closures and physical distancing measures (Dineen et al., 2022; Ravens-Sieberer et al., 2022). Coping with this unprecedented situation and complying with heavy restrictions can be particularly challenging, especially for adolescents who are in need of social interactions beyond the family (Ravens-Sieberer et al., 2022). Since sustained stressors during developmentally sensitive periods can have long lasting effects (Courtney et al., 2020), it is important to examine the far reaching impact of the pandemic on children and adolescents’ quality of life and mental health (Coker et al., 2023; Rider et al., 2021).

Two meta-analyses of longitudinal studies with pre-pandemic data points showed a small but non-significant increase in mental health symptoms among children and adolescents in the early stages of the COVID-19 pandemic (Robinson et al., 2022; Sun et al., 2023). In the following months and years, evidence for increased mental health problems and lower quality of life has been accumulating, with girls and older adolescents being disproportionately affected (Madigan et al., 2023; Racine et al., 2021; Ravens-Sieberer et al., 2022). This may reflect the deleterious impact of prolonged and/or repeated periods of lockdown, restrictions, altered lifestyle and uncertainty about the future. Whether mental health and quality of life indicators have returned to pre-pandemic levels after the overall lifting of measures remains unknown.

The COVID-19 pandemic has revealed and exacerbated existing structural and social inequalities within societies, leading to disproportionate negative impacts on the health and wellbeing of disadvantaged populations (Seligman et al., 2021). Adults with low levels of education, low income, a history of migration or belonging to an ethnic minority experienced high risks of both direct (e.g., SARS-CoV-2 infection, severe COVID-19 disease, hospital admission and death), and indirect (e.g., stress, mental health issues, job insecurity, financial hardship) consequences of the pandemic (Holmberg et al., 2022; Seligman et al., 2021). The pandemic may also have amplified pre-existing social inequalities leading to health inequities in children and adolescents (Dragano et al., 2022; Mannheim et al., 2022; Saatci et al., 2021): specific dimensions of inequality (such as poverty, low parental education, immigrant background, or racial/ethnic minority) were shown to be risk factors for adverse mental health outcomes among children and adolescents (Reiss, 2013), before and during the COVID-19 pandemic (Ravens-Sieberer et al., 2022; Xiao et al., 2022). However, most studies have examined the independent impacts of unidimensional socioeconomic and demographic categorizations on youth outcomes (Patil et al., 2018). As a result, the interplay of social, economic, and demographic characteristics which shape their daily experiences and needs is not well understood (Dineen et al., 2022). Using an intersectional framework (Crenshaw, 1989) can lead to more accurate identification of subgroups at greater risk of developing adverse outcomes, while also informing effective and targeted prevention and treatment strategies (Jackson et al., 2016; Patil et al., 2018).

In this study, we aimed to investigate how demographic characteristics intersect with socioeconomic dimensions to shape the social patterning of quality of life and mental health in children and adolescents, two years into the COVID-19 pandemic.

## 2 Methods

### 2.1 Study design and setting

We used data from the SEROCoV-KIDS study, a prospective cohort study, which aims to assess the medium- and long-term impacts of the COVID-19 pandemic on the health and well-being of children and adolescents. The eligibility criteria were to be aged between 6 months and 17 years and to live in the canton of Geneva (Switzerland) (Dumont et al., 2022). Eligible children were identified from (1) random samples drawn from state registries provided by the Swiss Federal Office of Statistics, (2) families who were previously selected from random samples of the general population at state level for COVID-19 seroprevalence studies conducted by our group, and were invited to participate in the cohort (Stringhini et al., 2020, 2021a, 2021b). Enrollment into the cohort and baseline assessment occurred from December 2021 to April 2022. We analyzed these data with those from a seroprevalence study conducted between April and June 2022 on an age-stratified random sample of the Geneva general population, with similar inclusion criteria and data collection procedures (Zaballa et al., 2023).

At baseline, all participants were invited to perform a serological test (by blood drawing) to measure anti-SARS-CoV-2 antibodies. One parent (or referent adult) per family completed one questionnaire on each of their participating children and one questionnaire on themselves and their household, with questions covering different domains such as socio-demographic data, physical and mental health, or lifestyle. From age 14 on, adolescents also answered a questionnaire about their daily lives and habits, and their pandemic-related experience. All questionnaires were completed online through the Specchio-COVID19 digital platform (Baysson et al., 2022), except for a few participants who preferred a paper version. In this case, the questionnaires were sent by post with a return envelope, then entered into the database by a research assistant.

### 2.2 Ethics

The Geneva Cantonal Commission for Research Ethics approved these studies (2020-00881, 2021-01973). Parents of participants and adolescents aged 14 years or older provided written informed consent. Children gave oral assent to participate.

### 2.3 Study population

**Fig. 1:**
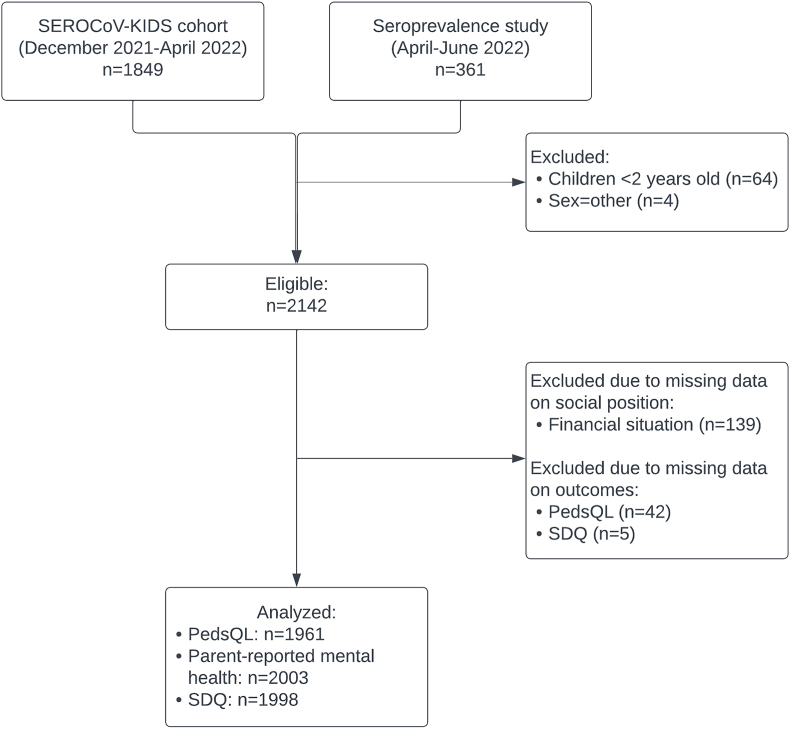
Flow Chart Legend: PedsQL: Pediatric quality of life inventory, SDQ: Strengths and difficulties questionnaire.

Given that our objective was to describe the social patterning of mental health and quality of life rather than to examine predictors or trends, we only used baseline data to conduct a cross-sectional analysis. We included all participants aged 2–17 years, and excluded four children whose sex was declared as “other”, due to insufficient sample size for intersectional analyses (Fig. 1). Children aged 6–23 months (n = 64) were not included because the scales we used are not validated before the age of 2 years.

### 2.4 Measures

All measures were parent-reported, except for health-related quality of life in participants aged 14 to 17, which was self-reported.

#### 2.4.1 Sociodemographic variables

We investigated the following sociodemographic dimensions: sex, age, immigrant background, parental education and financial situation. Sex was reported as male vs female. Age was calculated by taking the difference between the date of questionnaire completion and the date of birth, and categorized as 2–5, 6–11, and 12–17 years, in order to capture age-specific conditions of young children, children, and adolescents. These groups are consistent with the Swiss school system, where children start primary school at the age of 6 and secondary school at the age of 12. We classified children as having a Swiss background when they had at least one parent born in Switzerland, and as non-Swiss when both parents, or their referent parent in the case of single parenthood, were born abroad (hereafter referred to as “Swiss” and “non-Swiss”) (Ivert et al., 2020). We hypothesized that families with at least one parent born in Switzerland would have greater social support, stable legal status and better access to resources (e.g., housing, employment, services) than children with two foreign-born parents. This variable should therefore be considered as an effort to capture a social position affecting possibilities and life trajectories (Zettermark et al., 2021). Educational level was classified as low, middle or high based on the International Standard Classification of Education (2011) levels 0–2, 3–4 and 5–8, respectively (International Standard Classification of Education, 2023). We then considered the highest educational level (educational level of the referring parent in case of single parenthood), classified as high vs low/middle. The household financial situation was considered as poor when unexpected expenses could put the household into financial difficulties or when household members were not able to cover their expenses without external support, and good otherwise. The parents of 139 children chose the option "I prefer not to say" to answer the question about their financial situation.

#### 2.4.2 Health-related quality of life

Health-related quality of life (HRQoL) is a subjective and multidimensional construct that includes physiological, psychological, social and functional aspects of health and wellbeing. Adolescents aged 14 years and older and parents of children aged 2–13 years completed the French version of the Pediatric Quality of Life Inventory (PedsQL) Short Form (Chan et al., 2005). The 15 items were linearly transformed on a scale between 0 and 100 and averaged, with a higher score indicating a better HRQoL. Internal consistency was good (α = 0.88). In a complementary analysis, physical and psychosocial functioning were further assessed using the corresponding subscales (α = 0.90 and α = 0.85, respectively). Poor HRQoL, physical or psychosocial functioning were defined using thresholds compatible with a severe health condition (Varni et al., 2003).

#### 2.4.3 Mental health

Parent-reported mental health was assessed from the question “In general, how would you rate your child's mood?”, adapted from the Pediatric Global Health (PGH-7) Items, Parent-Proxy Report Form (Forrest et al., 2014). Responses were based on a five-point scale and were dichotomized into good (“very good” or “good”) vs poor (“fair”, “poor” or “very poor”).

Children's mental health difficulties were also measured using the French version of the Strengths and Difficulties Questionnaire (SDQ), a 25-item scale covering five domains: four "difficulty" domains (hyperactivity/inattention, emotional, conduct, peer relationship) and one "strength" domain (prosocial behavior) (Goodman, 1997; Shojaei et al., 2009). The total difficulties score was computed as the sum of the first four domains (range 0–40, with higher scores suggesting more significant problems). A low prosocial score indicated poor prosocial behavior. The existence of mental health difficulties was defined according to published clinical thresholds corresponding to the 90th percentile of the score distribution based on United Kingdom normative data (Goodman, 1997). As a complementary analysis, and following the recommendations for community samples (Goodman et al., 2010), we calculated the internalizing score by adding the emotional and peer problems subscales, and the externalizing score by combining the conduct problems and hyperactivity subscales. These scores were then dichotomized using cut-offs defined as the sum of the clinical cut-offs for the corresponding subscales. Internal consistency in this sample was good for the total difficulties score (α = 0.81), acceptable for externalizing problems (α = 0.78) and prosocial behaviors (α = 0.74) but questionable for internalizing problems (α = 0.67).

### 2.5 Statistical analyses

The dimensions of social position and outcomes were described as frequencies and percentages. Binomial confidence intervals (CIs) were calculated for outcome proportions. We then conducted an intersectional Multilevel Analysis of Individual Heterogeneity and Discriminatory Accuracy (MAIHDA) to examine health outcomes at the intersection of multiple social identities. This method for quantitative inter-categorical intersectionality research provides a socio-demographic mapping of poor quality of life and mental health among children and adolescents and quantifies the magnitude of any observed inequities (Axelsson Fisk et al., 2018; Evans, 2019a; Evans et al., 2018; Merlo, 2018).

Social strata were constructed for each of the possible unique combinations of sex, age, immigrant background, parental education and financial situation, resulting in a total of 48 strata (Fig. S1). There was substantial variability in strata sample size (range n = 3–211), and most had reasonable sample sizes (39/48 strata with more than 10 participants). We then fitted sequential multilevel models for each outcome in which children (level 1) were nested within intersectional social strata (level 2). Social positions were therefore treated as contextual-level variables (Beccia et al., 2021). Model 1 consisted of a *simple intersectional model*, i.e., a null model with random intercepts for social strata. Model 2 was an *intersectional interaction model*, which additionally included as fixed effects all variables used to define the intersectional strata. The fixed effects of each of those predictors, quantified by odds ratios (ORs), represent the main/additive effects of the specific category across all intersections (non-intersectional effects). (Moreno-Agostino et al., 2023).

We calculated two measures of general (sample-level) intersectionality effects. First, the Variance Partition Coefficient (VPC) in Model 1 quantifies the proportion of the total variance in the outcome attributable to differences across strata, while in Model 2 it corresponds to the proportion of the total variance that is unexplained by additive effects and potentially attributable to interaction effects (Evans, 2019b). The higher the VPC, the greater the similarities in outcomes among individuals within strata and the greater the differences across strata (Axelsson Fisk et al., 2018). It can therefore be interpreted as a measure of discriminatory accuracy (DA), or the ability of the model to correctly discriminate between people presenting or not the outcome of interest (Axelsson Fisk et al., 2018; Evans & Erickson, 2019). Second, the Proportional Change in Variance (PCV) reflects the proportion of the between-stratum variance from the null model that is accounted for by the inclusion of additive effects in Model 2.

We also obtained specific (stratum-level) intersectionality measures. First, the predicted proportions of the outcome estimates for each stratum were obtained from the simple intersectional models. They provided a detailed map of HRQoL and mental health inequalities, and helped identify specific strata with particularly positive or negative outcomes. Second, we calculated strata-level residuals in the intersectional interaction models, as the difference between the predicted outcome estimates for each stratum due to both main effects and interaction effects and expected estimates due to main effects only (Keller et al., 2023). Strata-level residuals represent the proportion of the outcome attributable to interaction (Moreno-Agostino et al., 2023).

Only participants who were assignable to social strata were included in the main analysis. A total of 139 participants (6.5%) could not be assigned to a stratum, due to missing data on their financial situation. Their characteristics are described in Table S1. We performed a sensitivity analysis by allocating all participants with missing data on financial situation to the poor financial situation group, and then to the good financial situation group. Participants were further excluded when outcome data were missing (range: n = 0 to n = 42).

All MAIHDA models were run in MLwiN 3.06 called from Stata 15.0 using the runmlwin command (Charlton et al.; Leckie & Charlton, 2013), following previously developed procedure and code (Axelsson Fisk et al., 2018; Evans, 2019a). We fitted the intersectional MAIHDA models using Bayesian Markov chain Monte Carlo (MCMC) procedures, specifying diffuse priors, a burn-in period of 5000 iterations and a total length of 50,000 iterations (with thinning every 50 iterations). Quasi-likelihood methods were used to provide the MCMC procedure with initialization values. Posterior samples were checked by visual assessments of trace plots. Stratum-specific values were obtained by transformation of the output from the logistic scale to the probability scale, and 95% credible intervals (CrI) were constructed using the 2.5 and 97.5 percentiles of the posterior distribution. Figures displaying stratum-specific predicted proportions were plotted using R (version 4.2.2) and the ggplot2 package.

## 3 Results

### 3.1 Sociodemographic characteristics

Among 2142 eligible participants, 49.5% were girls, 18.5%, 44.0% and 37.5% were young children (2–5 years old), children (6–11 years old) and adolescents (12–17 years old), respectively (Fig. 1, Table 1). The majority were living in households with at least one parent born in Switzerland, a high educational level and a good financial situation. Low HRQoL was reported in 11.5% (95% CI 10.1–12.9) of children and adolescents, with large variations depending on the dimension (physical or psychosocial). Regardless of the mental health outcome considered, there were small variations in the percentage of participants with poor mental health, ranging from 4% to 7%. Participants born to parents with a low education level and a poor financial situation had a higher frequency of all adverse outcomes (Table S2). Adolescents had more often a poor HRQoL and a poor parent-reported mental health compared to younger children.

**Table 1:** Characteristics of the analytical sample of children and adolescents.

	Analytical sample (n = 2003)^a^ n/N (%)
**Dimensions of social position**	
**Sex**	
Male	1017/2003 (50.8)
Female	986/2003 (49.2)
**Age (years)**	
2-5	375/2003 (18.7)
6-11	878/2003 (43.8)
12-17	750/2003 (37.5)
**Immigrant background**	
Swiss	1219/2003 (60.9)
Non-Swiss	784/2003 (39.1)
**Parental education**	
High	1666/2003 (83.2)
Low	337/2003 (16.8)
**Financial situation**	
Good	1624/2003 (81.1)
Poor	379/2003 (18.9)
**Outcomes**	
**Health-related quality of life** (PedsQL)^b^	
Poor health-related quality of life	224/1961 (11.4)
Poor physical health-related quality of life	126/1961 (6.4)
Poor psychosocial health-related quality of life	401/1961 (20.5)
**Poor parent-reported mental health**	136/2003 (6.8)
**Mental health difficulties** (SDQ)^b^	
Mental health difficulties	101/1998 (5.1)
Internalizing problems	87/1998 (4.4)
Externalizing problems	94/1998 (4.7)
Poor prosocial behavior	99/1998 (5.0)

PedsQL: Pediatric Quality of Life Inventory; SDQ: Strengths and Difficulties Questionnaire.

a The analytical sample corresponds to the eligible sample after exclusion of 139 participants who chose the option “I prefer not to say” to the question on financial situation, considered as missing data in this analysis.

b There were a few missing data for the PedsQL (n = 42) and SDQ (n = 5) scales, due to incomplete questionnaires filled out on paper and/or missing adolescent questionnaires.

### 3.2 Intersectional MAIHDA: general intersectionality effects

The degree of clustering into intersectional strata before including fixed effects, quantified by VPC, explained from 2.4% to 25.3% of the total variation in outcomes within the sample (Table 2, Table 3). In other words, the discriminatory accuracy of the variables defining the intersections was large for physical HRQoL (VPC = 23.4%) and parent-reported mental health (VPC = 25.3%), and was low for the total SDQ score (VPC = 2.4%). Between-stratum variance, VPCs and PCVs from the intersectional interaction models indicated that much, but not all, of this observed stratum-level variation was due to the additive effects of sex, age, migrant status, parental education and financial situation, except for mental health difficulties (PCV = 18.1%) and internalizing problems (PCV = 49.3%), where most of the variation was due to intersectional interactions.

**Table 2:** Logistic MAIHDA model results for health-related quality of life and mental health.

	Poor health-related quality of life (n = 1961)	Poor parent-reported mental health (n = 2003)	Mental health difficulties (n = 1998)
	Estimate (95%CrI)	Estimate (95%CrI)	Estimate (95%CrI)
**Simple intersectional model**			
*Random-effects*			
Between-stratum variance	0.69 (0.32–1.26)	1.18 (0.51–2.26)	0.08 (0.001–0.47)
VPC (%)	16.8 (8.9–27.8)	25.3 (13.3–40.8)	2.4 (0.0–12.6)
**Intersectional interaction model**			
*Fixed-effects odds ratios*			
Sex			
Male	Ref.	Ref.	Ref.
Female	1.32 (0.80–2.18)	1.20 (0.74–1.87)	0.70 (0.43–1.06)
Age (years)			
2–5	0.69 (0.32–1.30)	**0.33 (0.10–0.73)**	1.58 (0.84–2.70)
6–11	Ref.	Ref.	Ref.
12–17	1.68 (0.93–2.80)	**3.40 (2.06–5.50)**	0.85 (0.47–1.37)
Immigrant background			
Swiss	Ref.	Ref.	Ref.
Non-Swiss	1.20 (0.71–1.90)	0.74 (0.45–1.11)	**0.64 (0.36–0.99)**
Parental education			
High	Ref.	Ref.	Ref.
Low	1.37 (0.79–2.15)	1.22 (0.72–1.98)	**2.10 (1.16–3.36)**
Financial situation			
Good	Ref.	Ref.	Ref.
Poor	**2.48 (1.47–3.97)**	**2.66 (1.69–4.17)**	1.41 (0.81–2.22)
*Random effects*			
Between-stratum variance	0.31 (0.08–0.71)	0.09 (0.001–0.47)	0.07 (0.001–0.29)
VPC (%)	8.5 (2.4–17.8)	2.6 (0.04–12.5)	2.0 (0.03–8.1)
PCV (%)	53.5%	92.1%	18.1%
Bayesian DIC	1314.20	915.93	792.80

CrI: Credible interval, DIC: deviance information criterion, PCV: proportional change in variance, VPC: variance partition coefficient.

Numbers in bold are statistically significant.

**Table 3:** Logistic MAIHDA model results for the subscales of health-related quality of life and mental health difficulties.

	Health-related quality of life (PedsQL)		Mental health difficulties (SDQ)		
	Poor physical HRQoL (n = 1961)	Poor psychosocial HRQoL (n = 1961)	Internalizing problems (n = 1998)	Externalizing problems (n = 1998)	Poor prosocial behavior (n = 1998)
	Estimate (95%CrI)	Estimate (95%CrI)	Estimate (95%CrI)	Estimate (95%CrI)	Estimate (95%CrI)
**Simple intersectional model**					
*Random-effects*					
Between-stratum variance	1.03 (0.44–2.03)	0.37 (0.15–0.72)	0.59 (0.13–1.26)	0.32 (0.03–0.89)	0.54 (0.14–1.11)
VPC (%)	23.4 (11.8–38.2)	10.0 (4.3–18.0)	14.7 (3.8–27.7)	8.6 (0.8–21.4)	13.8 (4.1–27.0)
**Intersectional interaction model**					
*Fixed-effects odds ratios*					
Sex					
Male	Ref.	Ref.	Ref.	Ref.	Ref.
Female	1.71 (0.83–2.92)	1.15 (0.83–1.49)	1.69 (0.88–2.73)	**0.42 (0.25–0.66)**	0.71 (0.41–1.10)
Age (years)					
2–5	0.79 (0.34–1.53)	**0.46 (0.29–0.69)**	0.78 (0.28–1.67)	1.36 (0.74–2.33)	**5.55 (2.81–9.99)**
6–11	Ref.	Ref.	Ref.	Ref.	Ref.
12–17	1.58 (0.78–2.64)	1.27 (0.92–1.71)	1.91 (0.96–3.53)	0.62 (0.35–1.01)	1.99 (0.96–3.44)
Immigrant background					
Swiss	Ref.	Ref.	Ref.	Ref.	Ref.
Non-Swiss	1.39 (0.75–2.30)	0.92 (0.67–1.22)	1.35 (0.71–2.58)	0.81 (0.47–1.26)	0.82 (0.47–1.26)
Parental education					
High	Ref.	Ref.	Ref.	Ref.	Ref.
Low	1.64 (0.79–2.88)	1.08 (0.77–1.47)	**2.01 (1.01–3.59)**	1.45 (0.75–2.43)	1.23 (0.63–2.08)
Financial situation					
Good	Ref.	Ref.	Ref.	Ref.	Ref.
Poor	**2.65 (1.36–4.56)**	**2.10 (1.52–2.85)**	1.53 (0.76–2.73)	1.74 (0.96–2.71)	1.10 (0.55–1.83)
*Random effects*					
Between-stratum variance	0.39 (0.01–1.19)	0.06 (0.00–0.22)	0.30 (0.001–1.0)	0.04 (0.001–0.27)	0.11 (0.001–0.44)
VPC (%)	9.9 (0.2–26.6)	1.6 (0.04–6.4)	7.9 (0.03–23.6)	1.3 (0.01–7.6)	3.0 (0.02–11.9)
PCV (%)	62.1%	84.9%	49.3%	86.0%	80.2%
Bayesian DIC	886.63	1931.11	694.84	742.53	758.36

CrI: Credible interval, DIC: deviance information criterion, HRQoL: health-related quality of life, PCV: proportional change in variance, PedsQL: Pediatric Quality of Life Inventory, SDQ: Strengths and Difficulties Questionnaire, VPC: variance partition coefficient.

Numbers in bold are statistically significant.

We identified heterogeneous and outcome-specific social patterns of HRQoL and mental health (Table 2, Table 3). A poor financial situation was associated with all dimensions of low HRQoL and parent-reported poor mental health, with a similar adverse impact across most strata. Young children were less likely to have a low psychosocial HRQoL and poor parent-reported mental health, and more likely to have poor prosocial behaviors than children aged 6–11 years, while strata including adolescents were more prone to poor parent-reported mental health. Migrant status was not associated with any outcome, except for mental health difficulties, with lower odds among participants with two foreign-born parents. Girls were less likely to have externalizing problems (Table 3). Low parental education was associated with increased odds of mental health difficulties and internalizing problems (Table 3). In general, the sensitivity analyses provided similar estimates and interpretations. However, due to the narrower 95% CrIs, a few estimates became either significant or non-significant (Table 4). For instance, when all individuals with missing data were reclassified as having either poor or good financial situation, being an adolescent and low parental education were found to be risk factors for poor HRQoL.

**Table 4:** Sensitivity analysis showing the logistic MAIHDA model results after allocating all participants with missing data on financial situation to the poor or good financial situation group.

	Poor HRQoL (n = 2096)	Poor HRQoL (n = 2096)	Poor parent-reported mental health (n = 2142)	Poor parent-reported mental health (n = 2142)	Mental health difficulties (n = 2135)	Mental health difficulties (n = 2135)
	Allocation to the **poor** financial situation group	Allocation to the **good** financial situation group	Allocation to the **poor** financial situation group	Allocation to the **good** financial situation group	Allocation to the **poor** financial situation group	Allocation to the **good** financial situation group
	Estimate (95%CrI)	Estimate (95%CrI)	Estimate (95%CrI)	Estimate (95%CrI)	Estimate (95%CrI)	Estimate (95%CrI)
**Simple intersectional model**						
*Random-effects*						
Between-stratum variance	0.67 (0.30–1.33)	0.67 (0.32–1.20)	1.02 (0.40–2.12)	0.95 (0.38–1.94)	0.09 (0.001–0.42)	0.11 (0.001–0.47)
VPC (%)	16.8 (8.3–28.7)	16.6 (8.8–26.7)	22.8 (10.8–39.1)	21.6 (10.3–37.1)	2.7 (0.0–11.4)	3.1 (0.0–12.5)
**Intersectional interaction model**						
*Fixed-effects odds ratios*						
Sex						
Male	Ref.	Ref.	Ref.	Ref.	Ref.	Ref.
Female	1.26 (0.79–1.96)	1.32 (0.81–1.98)	1.17 (0.73–1.81)	1.21 (0.82–1.74)	0.73 (0.43–1.12)	0.73 (0.46–1.09)
Age (years)						
2–5	0.71 (0.37–1.26)	0.68 (0.34–1.27)	**0.36 (0.11–0.80)**	**0.36 (0.12–0.75)**	1.60 (0.87–2.64)	1.60 (0.91–2.63)
6–11	Ref.	Ref.	Ref.	Ref.	Ref.	Ref.
12–17	**1.72 (1.02–2.73)**	**1.77 (1.08–2.83)**	**3.15 (1.86–5.05)**	**3.10 (1.98–4.79)**	0.84 (0.46–1.35)	0.85 (0.50–1.32)
Immigrant background						
Swiss	Ref.	Ref.	Ref.	Ref.	Ref.	Ref.
Non-Swiss	1.36 (0.84–2.09)	1.38 (0.84–2.12)	0.75 (0.47–1.14)	0.74 (0.49–1.11)	0.69 (0.41–1.04)	0.67 (0.41–1.03)
Parental education						
High	Ref.	Ref.	Ref.	Ref.	Ref.	Ref.
Low	**1.71 (1.04–2.66)**	1.63 (0.99–2.55)	1.33 (0.79–2.11)	1.25 (0.76–1.88)	**2.40 (1.36–3.85)**	**2.26 (1.33–3.46)**
Financial situation						
Good	Ref.	Ref.	Ref.	Ref.	Ref.	Ref.
Poor	**2.05 (1.27–3.20)**	**2.20 (1.35–3.42)**	**2.20 (1.38–3.49)**	**2.52 (1.58–3.78)**	1.18 (0.69–1.84)	1.41 (0.82–2.22)
*Random effects*						
Between-stratum variance	0.29 (0.08–0.65)	0.27 (0.07–0.64)	0.13 (0.002–0.47)	0.05 (0.001–0.25)	0.06 (0.001–0.34)	0.05 (0.001–0.26)
VPC (%)	7.9 (2.5–16.4)	7.4 (2.1–16.2)	3.6 (0.06–12.6)	1.4 (0.02–7.1)	1.8 (0.02–9.3)	1.4 (0.03–7.5)
PCV (%)	57.5%	59.6%	87.4%	95.0%	31.5%	58.2%
Bayesian DIC	1405.92	1406.15	984.93	981.75	829.14	827.51

CrI: Credible interval, DIC: deviance information criterion, HRQoL: health-related quality of life, PCV: proportional change in variance, VPC: variance partition coefficient.

Numbers in bold are statistically significant.

### 3.3 Intersectional MAIHDA: specific intersectionality effects

**Fig. 2:**
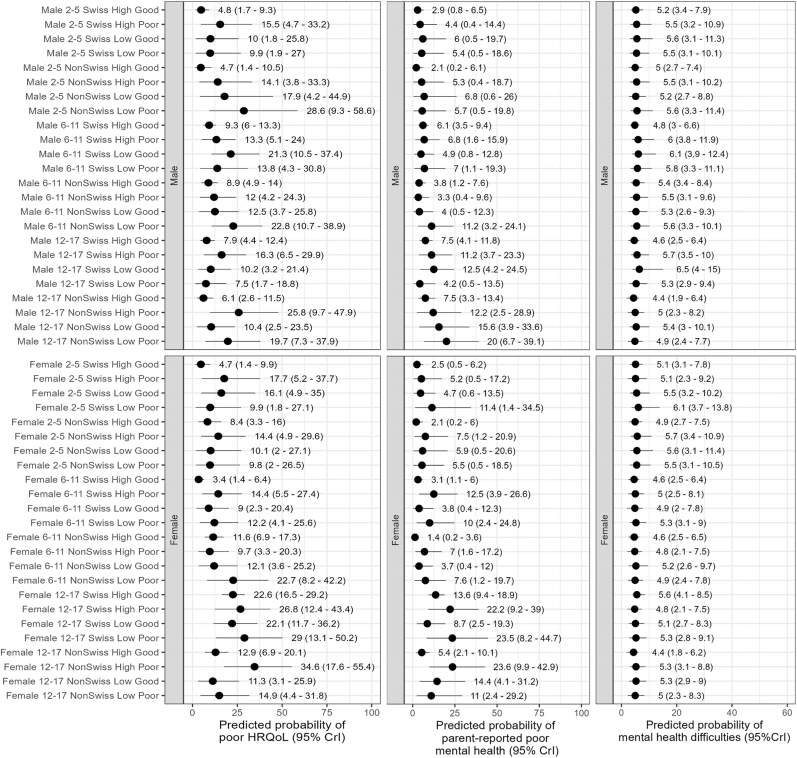
Social stratum-specific predicted proportions of health-related quality of life (left), parent-reported poor mental health (center) and mental health difficulties (right) Interpretation: This figure shows the stratum-specific predicted probability of each outcome obtained from the corresponding simple intersectional models.

**Fig. 3:**
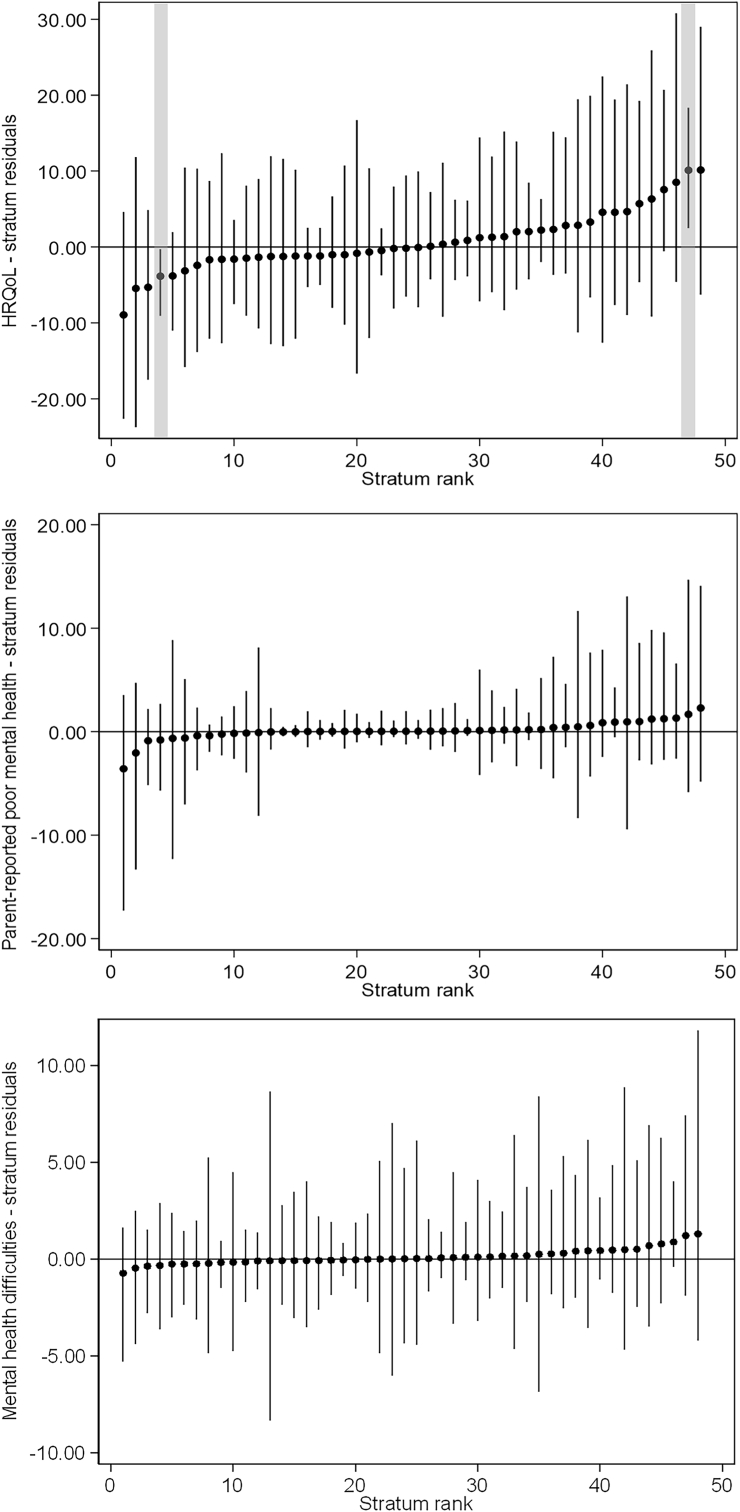
Estimated intersectional effects (stratum-level residuals) Interpretation: We present here strata-level residuals (attributable to intersectional interaction) and their corresponding 95% credible intervals for each stratum ranked from lowest to highest. Strata with significant intersectional effect (i.e., their 95% credible intervals do not overlap with 0) are highlighted in grey.

**Fig. 4:**
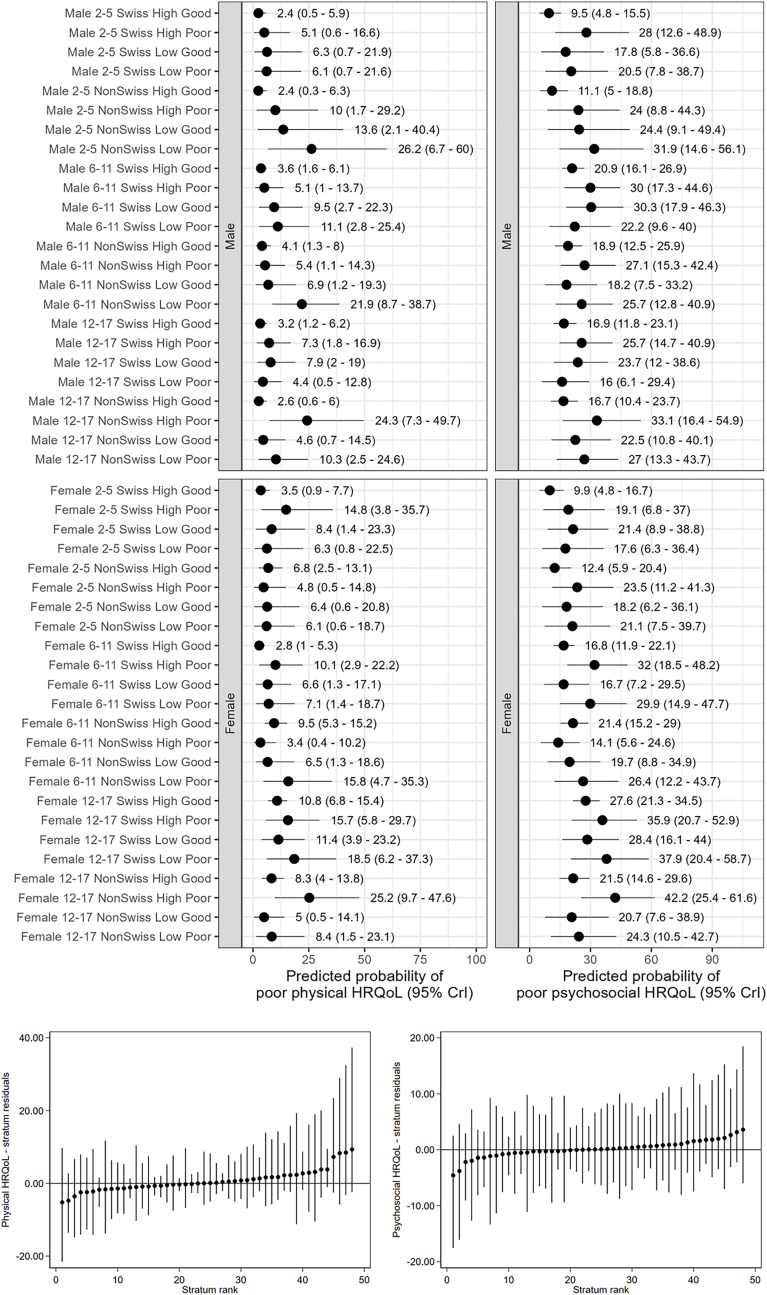
Social stratum-specific predicted proportions of physical and psychosocial health-related quality of life (top panel) and estimated intersectional effects (stratum residuals - bottom panel).

**Fig. 5:**
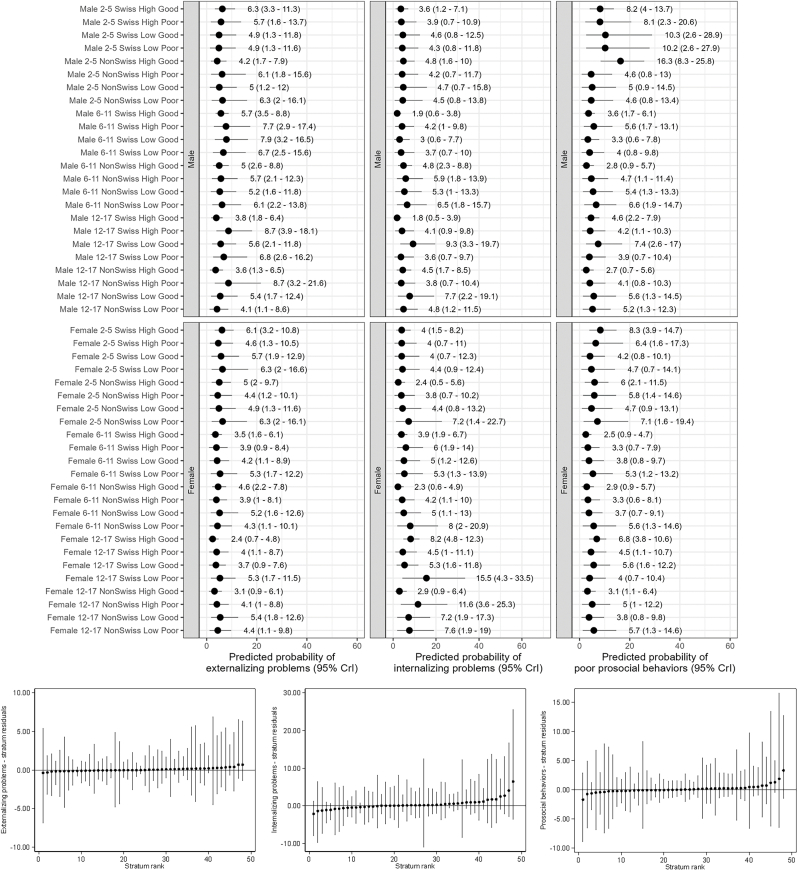
Social stratum-specific predicted proportions of externalizing problems, internalizing problems and prosocial behaviors (top panel) and estimated intersectional effects (stratum residuals - bottom panel).

Stratum-specific predicted proportions of outcomes further highlighted substantial heterogeneity across strata (Fig. 2, Fig. 4, Fig. 5). Poor HRQoL varied by a factor of 10, ranging from 3.4% for 6–11 years old Swiss girls with highly educated parents and no financial hardship to 34.6% for 12–17 years old non-Swiss girls with highly educated parents and financial hardship. Between-stratum variations in the predicted frequency of parent-reported poor mental health were also large (range 1.4%–23.6%), but those of mental health difficulties were not (range 4.4%–6.5%). Strata including participants with socioeconomic disadvantage, or conflicting socio-economic positions (e.g., high education and financial hardship), tended to have a higher predicted proportion of poor outcomes than their counterparts. Most differences were accounted for by the main/additive effects of the variables defining the strata, and almost all intersectional effects overlapped with zero (no significant effect) (Fig. 3, Figs. 4 and 5). Only two strata displayed significant interaction (i.e., their 95% credible intervals did not include 0), namely 6–11 and 12–17 years old Swiss girls with highly educated parents and good financial situation, who respectively had a lower than expected and higher than expected HRQoL (Fig. 3). The sensitivity analyses produced consistent findings, with predicted proportions of poor HRQoL slightly attenuated when all participants with missing data were allocated to the poor financial situation group (Fig. S2, Fig. S3, Fig. S4).

## 4 Discussion

### 4.1 Main findings

Intersectionality theory highlights the importance of the interplay of multiple socioeconomic and demographic dimensions in shaping individual well-being and health outcomes. In this study, we found evidence of meaningful intersectionality between sex, age, immigrant background, parental education and financial situation in structuring the social patterning of health-related quality of life and mental health among children and adolescents, about two years after the start of the COVID-19 pandemic. Intersectional strata involving adolescents and families with financial hardship showed substantially worse health-related quality of life and parent-reported poor mental health. Between-stratum variations in the predicted frequency of mental health difficulties were limited. Differences in the predicted values of all outcomes, but mental health difficulties, painted a more accurate and nuanced picture: they reflected large inequalities between strata, particularly among socio-economically disadvantaged populations.

### 4.2 Interpretation

The existing body of literature contains numerous instances highlighting the connections between specific socioeconomic factors and mental health issues during childhood and adolescence. A systematic review encompassing 55 observational studies involving children aged 4–18 years revealed that children from socioeconomically disadvantaged families were two to three times more likely to develop mental health problems compared to their counterparts from more advantaged backgrounds (Reiss, 2013). The association between socioeconomic indicators and mental health problems was observed in all age groups, even in early childhood, with the strength of the effect varying with age. Multiple dimensions were examined, including income, relative poverty, welfare benefit receipt, social class, parental educational attainment, and parental occupational status, with the latter two emerging as the strongest predictors of mental health problems among the pediatric population. Recent studies have further shown that other socioeconomic dimensions, such as food insecurity, unemployment, migration and belonging to racial/ethnic minority groups, were independently associated with disparities in child mental health and quality of life (Barbieri et al., 2022; Ravens-Sieberer et al., 2022; Xiao et al., 2022). However, existing research has largely overlooked the intricate complexities that arise from the intersections of multiple sociodemographic dimensions (Dineen et al., 2022; Reiss, 2013).

To our knowledge, in the pandemic context no study has assessed mental health inequalities among children and adolescents using an intersectional approach. Pre-pandemic, Kern et al. examined the interplay between immigration background, socioeconomic status, and gender on mental well-being across 33 European countries, using data from the cross-national 2017/2018 Health Behaviour in School-aged Children study (11-, 13-, and 15-year-old adolescents) (Kern et al., 2020). These factors only explained a modest fraction of variations in mental well-being. However, across all countries, members of any of the disadvantaged social groups had higher life dissatisfaction and psychosomatic complaints, in comparison to adolescents from advantaged groups, with no evidence for intersectional effects. Using a similar analytical approach based on the Add Health longitudinal survey (United States, last wave in 2008), Evans et al. showed meaningful between-strata inequalities in depression throughout adolescence and young adulthood (age range 11–34 years old), with women, racial/ethnic minorities, immigrants, and low-income strata experiencing elevated depression scores, adjusting for age (Evans & Erickson, 2019).

Intersectional inequalities during the third national lockdown have recently been reported in young adults (19–21 and 31 years old) from two British cohorts (Moreno-Agostino et al., 2023). Our results are in line with these findings, and further suggest that some patterns seem specific to the pediatric population. Indeed, some age-specific differences related to developmental stages are expected (Rider et al., 2021), such as a poorer mental health with increasing age (Thorisdottir et al., 2021). Gender-dependent variations in mental health emerge over time (Martel, 2013; Panchal et al., 2021), in particular during adolescence due to rapid biological and social changes. Inequalities are largely shaped by the family environment starting in early childhood (Cotton & Shim, 2022), with an interplay between pre-existing factors with a sustained impact (e.g., parental education) and factors potentially exacerbated by the pandemic (e.g., financial hardship). Interestingly, our findings are from a high-income country with universal health coverage and a relatively generous welfare system. Further, Geneva (Switzerland) was largely hit by the pandemic (Zaballa et al., 2023), but implemented relatively light restrictions, and limited school closures to the first wave (from March to May 2020). We did not identify a major impact of immigrant background on most outcomes. This could be explained by a particular migratory context in Geneva, with a large share of foreigners (41% of the cantonal population in 2022), who are frequently highly qualified because of the particular economic structure of the state, hosting a large number of international organizations and multinational companies, and favorable migrant health policies, according to the Migrant Integration Policy Index (MIPEX). (www.mipex.eu, 2023).

Most mental health disorders start in childhood and adolescence (Hafstad & Augusti, 2021). By recognizing and addressing the diversity among children, it is possible to better understand their needs and provide targeted support to help them thrive. The discriminatory accuracy of our models indicates good to very good differentiation between strata, except for mental health difficulties (poor), and externalizing problems (fair) (Axelsson Fisk et al., 2018). Along with the socio-demographic mapping of different mental health measures in our population, discriminatory accuracy is relevant to inform public policies and resource allocation (Persmark et al., 2019). To maximize effectiveness, interventions to address inequities in health-related quality of life should target specific intersectional strata involving adolescents and families with financial hardship. Poor discriminatory accuracy and limited predicted between-stratum variations were shown for mental health difficulties. This suggests that all social groups were susceptible to mental health difficulties, and that population-level strategies would be more appropriate. Therefore, a layered public health approach to address children's and adolescents' mental health could be considered, combining universal measures (universal screening, mental health promotion and prevention of mental health concerns, school-based mental health support for youth, political and economic measures to reduce poverty) and targeted interventions (screening high-risk populations, community-based parenting and family preventive interventions by providing tools to manage stress and challenges) (Rider et al., 2021). These principles align with the idea of proportionate universalism aiming to reduce health inequalities and improve health and well-being for all (Marmot et al., 2010).

### 4.3 Strengths and limitations

A major strength of this work is the use of the recently developed MAIHDA approach to investigate health-related quality of life and mental health inequalities in an under-studied population drawn from a population-based study. We brought together several dimensions of identity and social experiences identified by the literature, namely sex, immigrant background, education and financial situation, and considered age as an additional relevant factor due to the differences in experiences across age groups. This approach converges with the current movement toward precision public health, through improving the evidence base for health policy by increasing understanding of both health inequalities and individual heterogeneity (Persmark et al., 2019).

Our results should be interpreted in the light of their limitations. Our cross-sectional data provide a snapshot of inequalities at one time-point, coinciding with the time when all restrictions were being progressively lifted in Switzerland. Our results may not be generalizable to other pandemic periods, in particular lockdowns, or to other countries, but they provide important insights into the post-acute phase of the pandemic and help orient management strategies and political decisions. We will keep monitoring health-related quality of life and mental health in the cohort, and may cover this gap in the future by extending this approach to longitudinal data. Also, we could not directly assess pandemic-related evolutions in financial situation and outcomes, due to the lack of pre-pandemic data. Parent-reported outcome measures may not be a perfect reflection of a child's condition and should not be interpreted as a diagnosis of mental health. However, they are acceptable, well-validated and evidence-based screening tools, making them useful for population-based research studies (Anderson et al., 2020). They can also be integrated into primary care practice, helping to reduce mental health stigma and providing a forum for families to discuss and obtain appropriate referrals whenever necessary. The number of participants in some minoritized strata (e.g., high education and financial hardship) was small. Even though MAIHDA models provide conservative and reliable estimates for strata with small numbers via shrinkage (Evans & Erickson, 2019), this may have led to a lack of precision in estimates and ability to detect intersectional effects at some of these intersections which may be at risk. Sexual orientation may be an important factor to consider in future research on adolescents (Moreno-Agostino et al., 2023), but we did not take this dimension into account as it was not relevant for younger children. As our sample was drawn from cantonal administrative registries, we could not include undocumented migrants who face substantially different structural issues and usually experience more mental health difficulties. Finally, we cannot exclude a self-selection bias, with eligible families refusing to participate because of language barriers (all questionnaires were only provided in French), low education level or low health literacy.

## 5 Conclusion

We found evidence of considerable differences in adverse outcomes across social strata, thereby increasing our understanding of the dynamics of privilege and disadvantage that drive the production of health disparities in children and adolescents. If not properly addressed through policy and community-level interventions, the social crisis created by the COVID-19 pandemic may further increase health problems and health inequalities in the medium and long term. Future research should shed light on psychological resources, resilience factors, and coping strategies that helped children, adolescents and families navigate the pandemic, and determine if they are socially patterned.

## Contributions

Elsa Lorthe: Conceptualization, Funding acquisition, Investigation, Formal analysis, Visualization, Writing - Original Draft; Viviane Richard: Investigation, Data Curation, Writing - Review & Editing; Roxane Dumont: Investigation, Data Curation, Writing - Review & Editing; Andrea Loizeau: Investigation, Funding acquisition, Project administration, Writing - Review & Editing; Javier Perez-Saez: Validation, Data Curation, Writing - Review & Editing; Hélène Baysson: Investigation, Validation, Writing - Review & Editing; Maria-Eugenia Zaballa: Investigation, Validation, Writing - Review & Editing; Julien Lamour: Validation, Data Curation, Writing - Review & Editing; Nick Pullen: Validation, Data Curation, Visualization, Writing - Review & Editing; Stephanie Schrempft: Investigation, Validation, Writing - Review & Editing; Rémy P. Barbe: Conceptualization, Funding acquisition, Supervision, Writing - Review & Editing; Klara M. Posfay-Barbe: Conceptualization, Funding acquisition, Supervision, Writing - Review & Editing; Idris Guessous: Conceptualization, Funding acquisition, Supervision, Writing - Review & Editing; Silvia Stringhini: Conceptualization, Funding acquisition, Project administration, Supervision, Writing - Review & Editing; All authors read and approved the final manuscript.

## Presentation information

This study was presented as a poster at the 17th World Congress on Public Health, Rome, May 2–6, 2023.

## Abbreviated title

Intersectional analysis of child well-being.

## Funding

Federal Office of Public Health of Switzerland, Jacobs Foundation, General Directorate of Health in Geneva canton, and Private Foundation of the Geneva University Hospitals. The funders of the study had no role in the study design, data collection, data analysis, data interpretation, or writing of this article.

## Declaration of competing interest

The authors declare the following financial interests/personal relationships which may be considered as potential competing interests: KMPB is a member of the Advisory Boards for pneumococcal vaccine and varicella vaccine at MSD. All other authors declare that they have no competing interests.

## Data Availability

Data will be made available on request.
