# Supplementary material for: Socioeconomic conditions and children's mental health and quality of life during the COVID-19 pandemic: An intersectional analysis
Source: SSM Popul Health. 2023 Jul 23;23:101472. doi: 10.1016/j.ssmph.2023.101472 (PMC10407575; doi:10.1016/j.ssmph.2023.101472)

**Supplementary material**

**Socioeconomic conditions and children’s mental health and quality of life during the COVID-19 pandemic: an intersectional analysis**

Table S1: Characteristics of children and adolescents included and excluded from the main analysis due to missing data on financial situation

|  |  | Total sample  n/N (%) | Analyzed sample  n/N (%) | Non analyzed sample n/N (%) |
| --- | --- | --- | --- | --- |
| **Dimensions of social position** | |  |  |  |
| **Sex** | |  |  |  |
|  | Male | 1082/2142 (50.5) | 1017/2003 (50.8) | 65/139 (46.8) |
|  | Female | 1060/2142 (49.5) | 986/2003 (49.2) | 74/139 (53.2) |
| **Age (years)** | |  |  |  |
|  | 2-5 | 395/2142 (18.5) | 375/2003 (18.7) | 20/139 (14.4) |
|  | 6-11 | 943/2142 (44.0) | 878/2003 (43.8) | 65/139 (46.8) |
|  | 12-17 | 804/2142 (37.5) | 750/2003 (37.5) | 54/139 (38.8) |
| **Immigrant background** | |  |  |  |
|  | Swiss | 1292/2142 (60.3) | 1219/2003 (60.9) | 73/139 (52.5) |
|  | Non-Swiss | 850/2142 (39.7) | 784/2003 (39.1) | 66/139 (47.5) |
| **Parental education** | |  |  |  |
|  | High | 1778/2142 (83.0) | 1666/2003 (83.2) | 112/139 (80.6) |
|  | Low | 364/2142 (17.0) | 337/2003 (16.8) | 27/139 (19.4) |
| **Financial situation** | |  |  |  |
|  | Good | 1624/2003 (81.1) | 1624/2003 (81.1) | - |
|  | Poor | 379/2003 (18.9) | 379/2003 (18.9) | - |
| **Outcomes^a^** | |  |  |  |
| **Health-related quality of life** (PedsQL)^b^ | |  |  |  |
|  | Poor health-related quality of life | 240/2096 (11.5) | 224/1961 (11.4) | 16/135 (11.9) |
|  | Poor physical health-related quality of life | 140/2096 (6.7) | 126/1961 (6.4) | 14/135 (10.4) |
|  | Poor psychosocial health-related quality of life | 427/2096 (20.4) | 401/1961 (20.5) | 26/135 (19.3) |
| **Poor parent-reported mental health** | | 144/2142 (6.7) | 136/2003 (6.8) | 8/139 (5.8) |
| **Mental health difficulties** (SDQ)^b^ | |  |  |  |
|  | Mental health difficulties | 105/2135 (4.9) | 101/1998 (5.1) | 4/137 (2.9) |
|  | Internalizing problems | 90/2135 (4.2) | 87/1998 (4.4) | 3/137 (2.2) |
|  | Externalizing problems | 97/2135 (4.5) | 94/1998 (4.7) | 3/137 (2.2) |
|  | Poor prosocial behavior | 102/2135 (4.8) | 99/1998 (5.0) | 3/137 (2.2) |

PedsQL: Pediatric Quality of Life Inventory; SDQ: Strengths and Difficulties Questionnaire

^a^The analytical sample corresponds to the eligible sample after exclusion of 139 participants with missing data on financial situation.

^b^There were a few missing data for the PedsQL (n=42) and SDQ (n=5) scales, due to incomplete questionnaires filled out on paper and/or missing adolescent questionnaires.

Table S2: Outcomes by socio-demographic dimension

|  | | Poor health-related quality of life | Poor parent-reported mental health | Mental health difficulties |
| --- | --- | --- | --- | --- |
|  | | n/N (%) | n/N (%) | n/N (%) |
| **Sex** | |  |  |  |
|  | Male | 103/1057 (9.7) | 66/1082 (6.1) | 61/1079 (5.7) |
|  | Female | 137/1039 (13.2) | 78/1060 (7.4) | 44/1056 (4.2) |
| **Age (years)** | |  |  |  |
|  | 2-5 | 24/391 (6.1) | 6/395 (1.5) | 26/391 (6.7) |
|  | 6-11 | 90/943 (9.5) | 42/943 (4.5) | 45/940 (4.8) |
|  | 12-17 | 126/762 (16.5) | 96/804 (11.9) | 34/804 (4.2) |
| **Immigrant background** | |  |  |  |
|  | Swiss | 137/1271 (10.8) | 96/1292 (7.4) | 71/1291 (5.5) |
|  | Non-Swiss | 103/825 (12.5) | 48/850 (5.7) | 34/844 (4.0) |
| **Parental education** | |  |  |  |
|  | High | 174/1747 (10.0) | 108/1778 (6.1) | 73/1774 (4.1) |
|  | Low | 66/349 (18.9) | 36/364 (9.9) | 32/361 (8.9) |
| **Financial situation** | |  |  |  |
|  | Good | 150/1592 (9.4) | 88/1624 (5.4) | 74/1620 (4.6) |
|  | Poor | 74/369 (20.1) | 48/379 (12.7) | 27/378 (7.1) |

We present here unadjusted line percentages.

Figure S1: Construction of social strata in this intersectional analysis


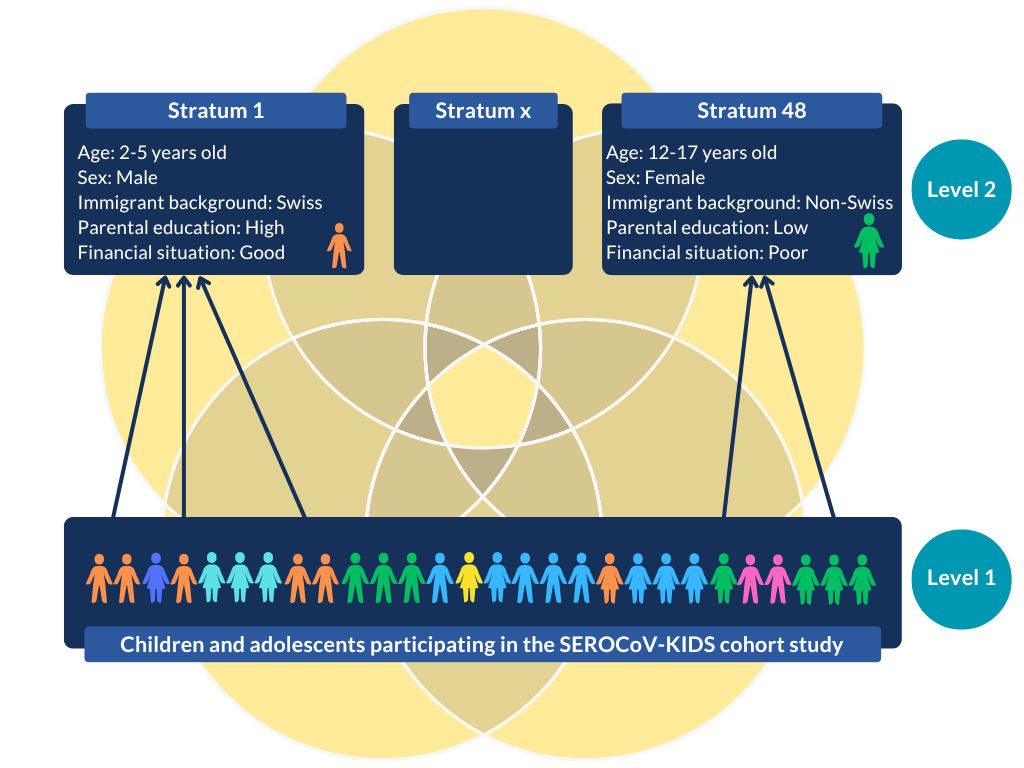


Figure S2: Sensitivity analysis displaying the social stratum-specific predicted proportions of poor HRQoL after allocating all participants with missing data on financial situation to the poor (left) or good (right) financial situation group


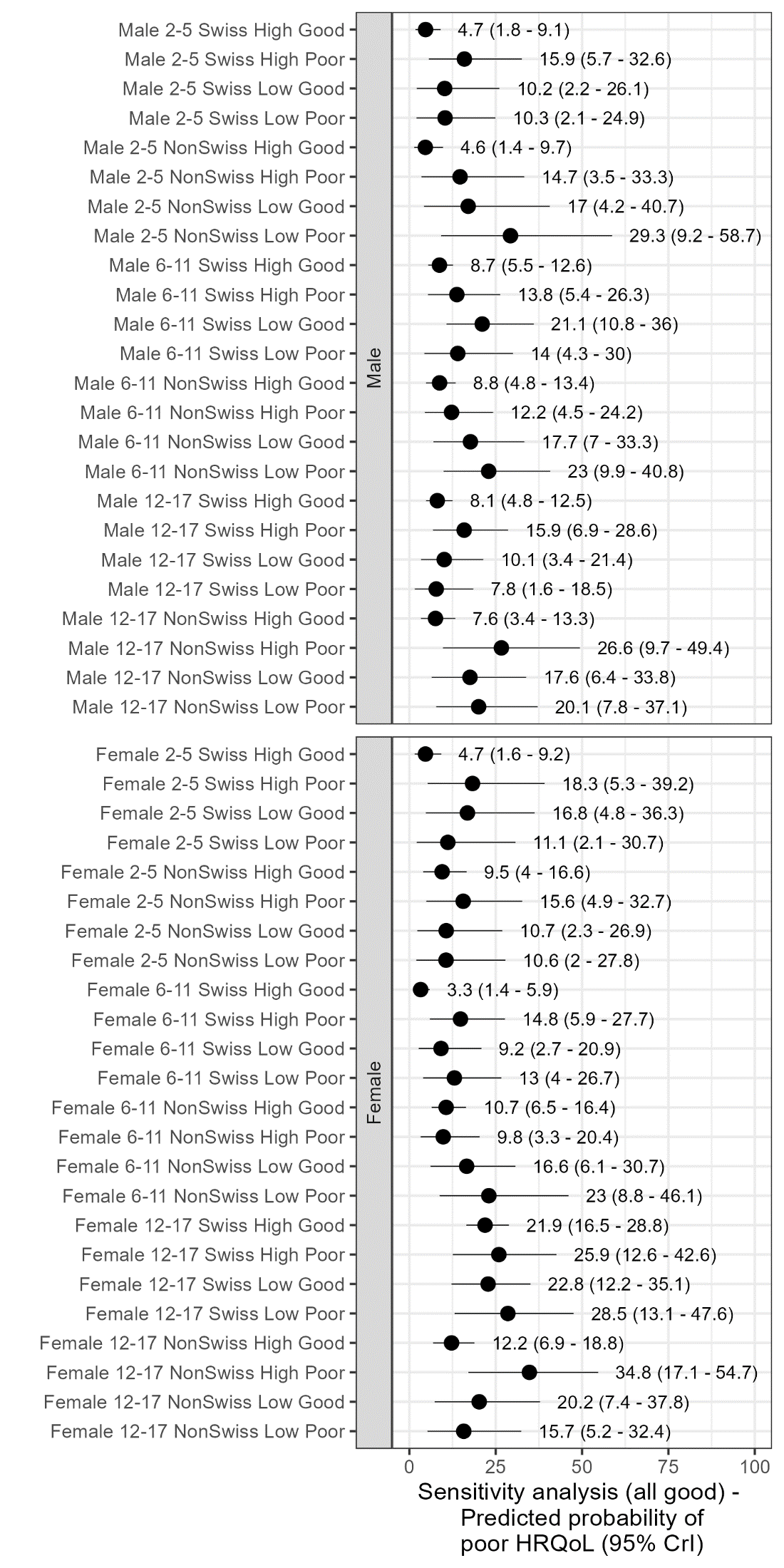

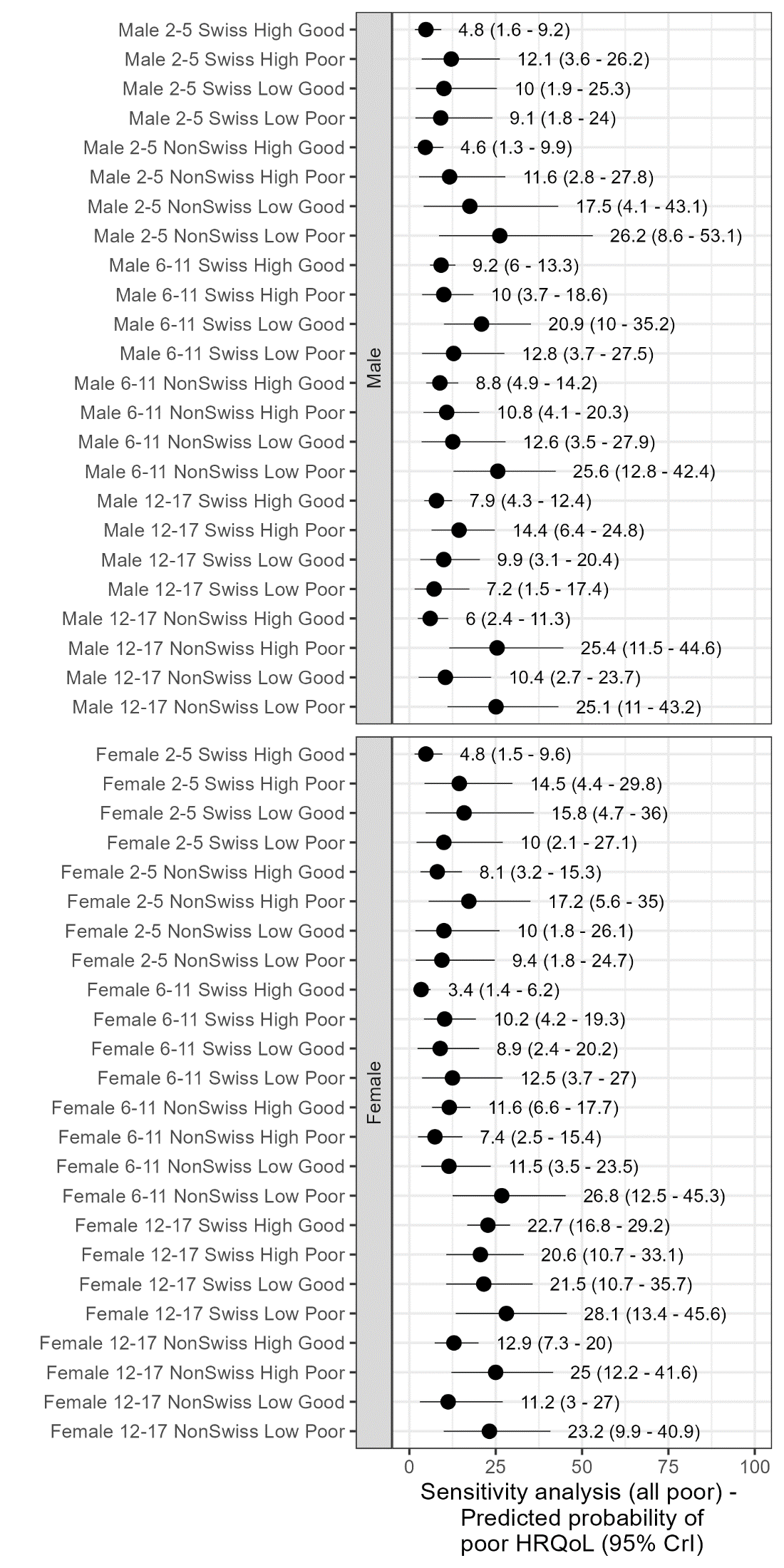


Figure S3: Sensitivity analysis displaying the social stratum-specific predicted proportions of poor parent-reported mental health after allocating all participants with missing data on financial situation to the poor (left) or good (right) financial situation group


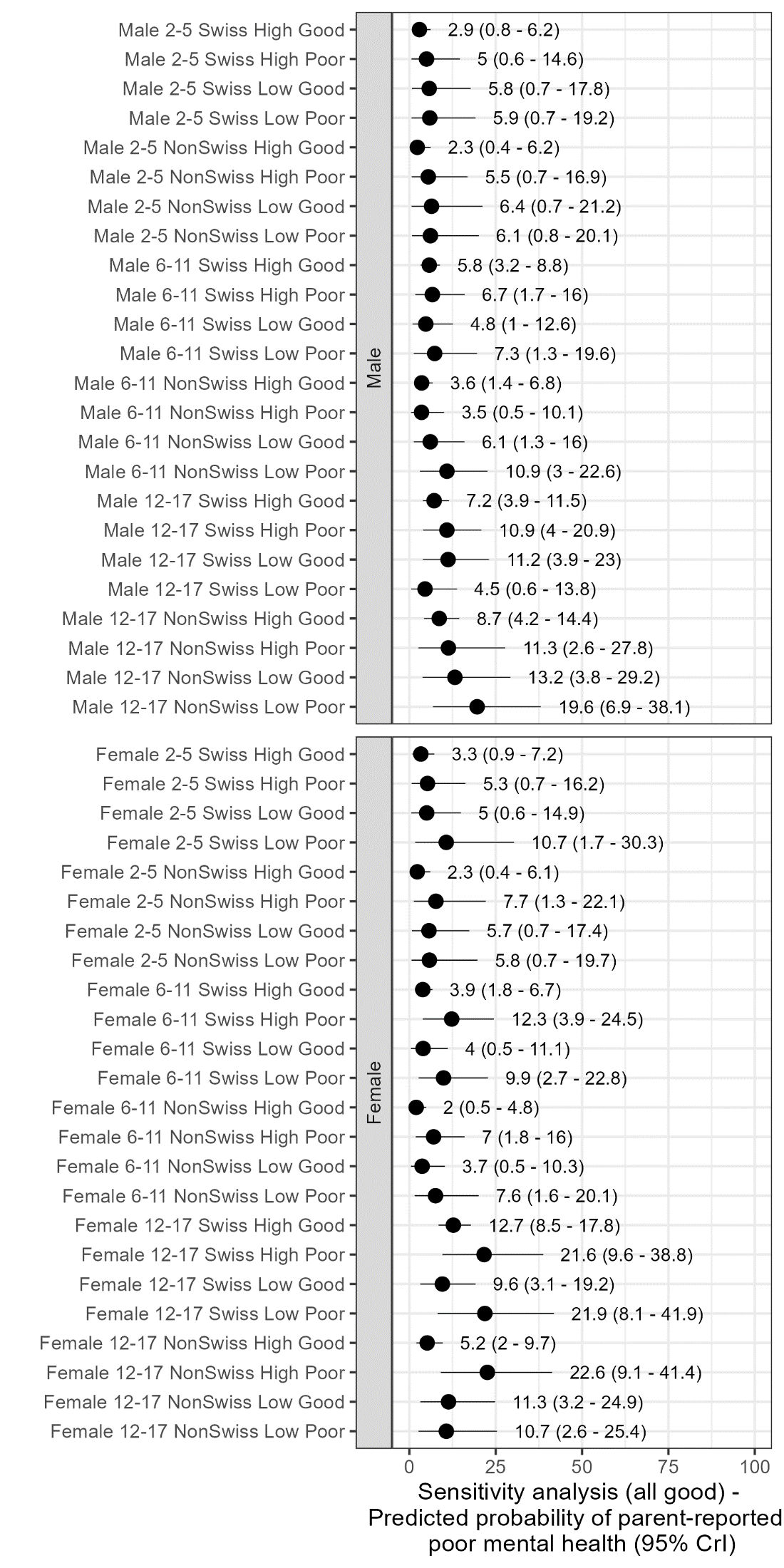

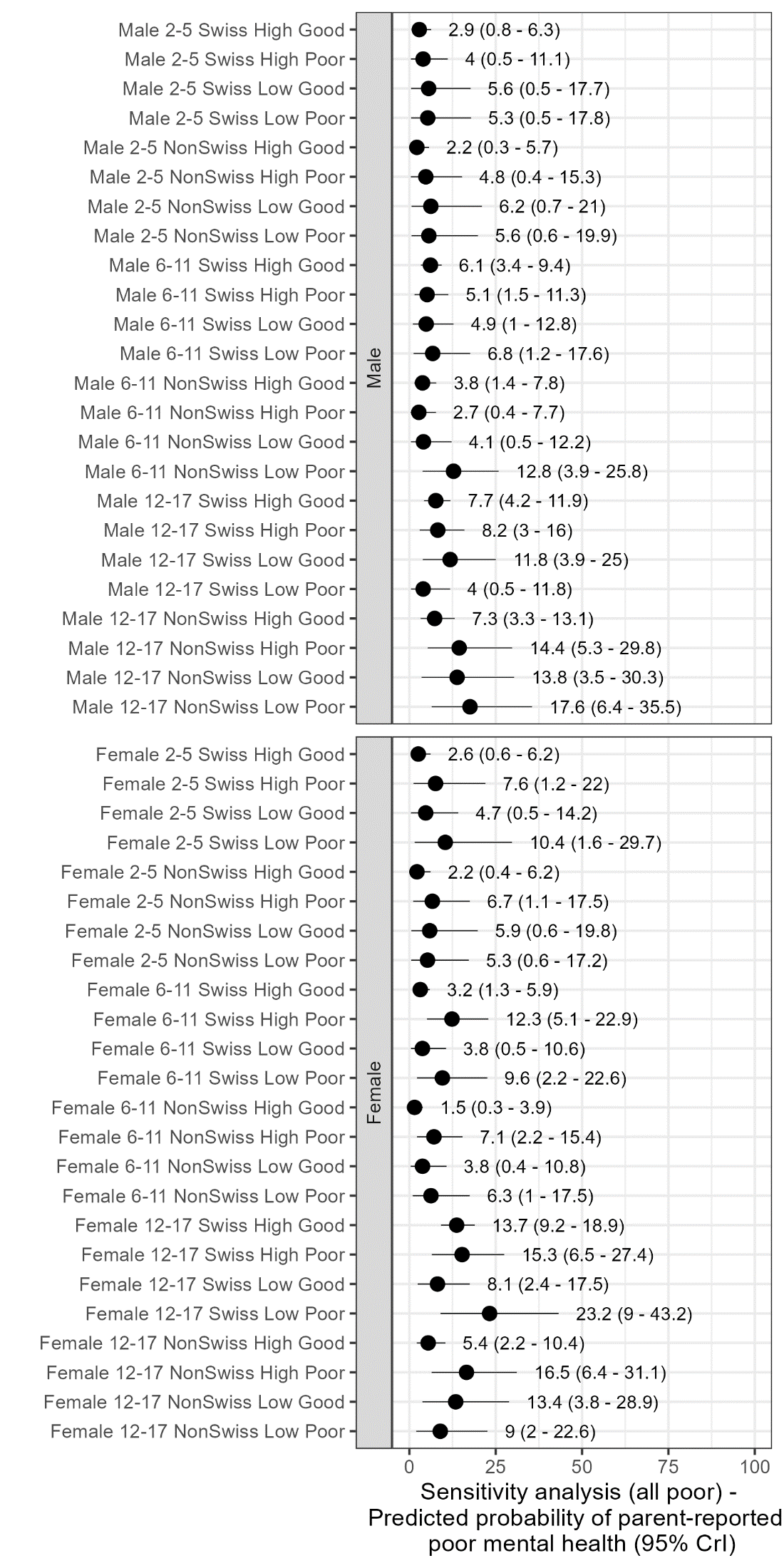


Figure S4: Sensitivity analysis displaying the social stratum-specific predicted proportions of mental health difficulties after allocating all participants with missing data on financial situation to the poor (left) or good (right) financial situation group


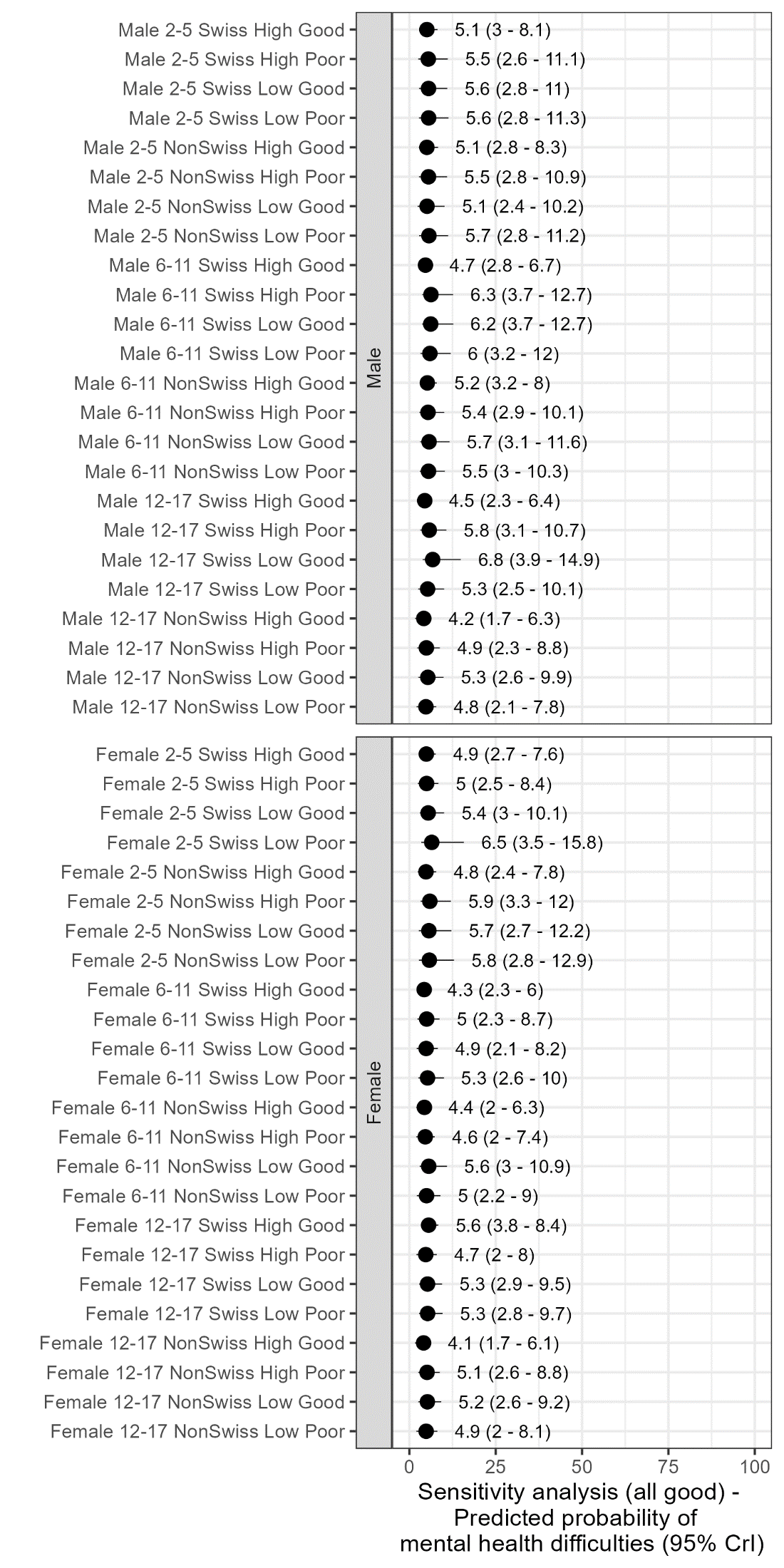

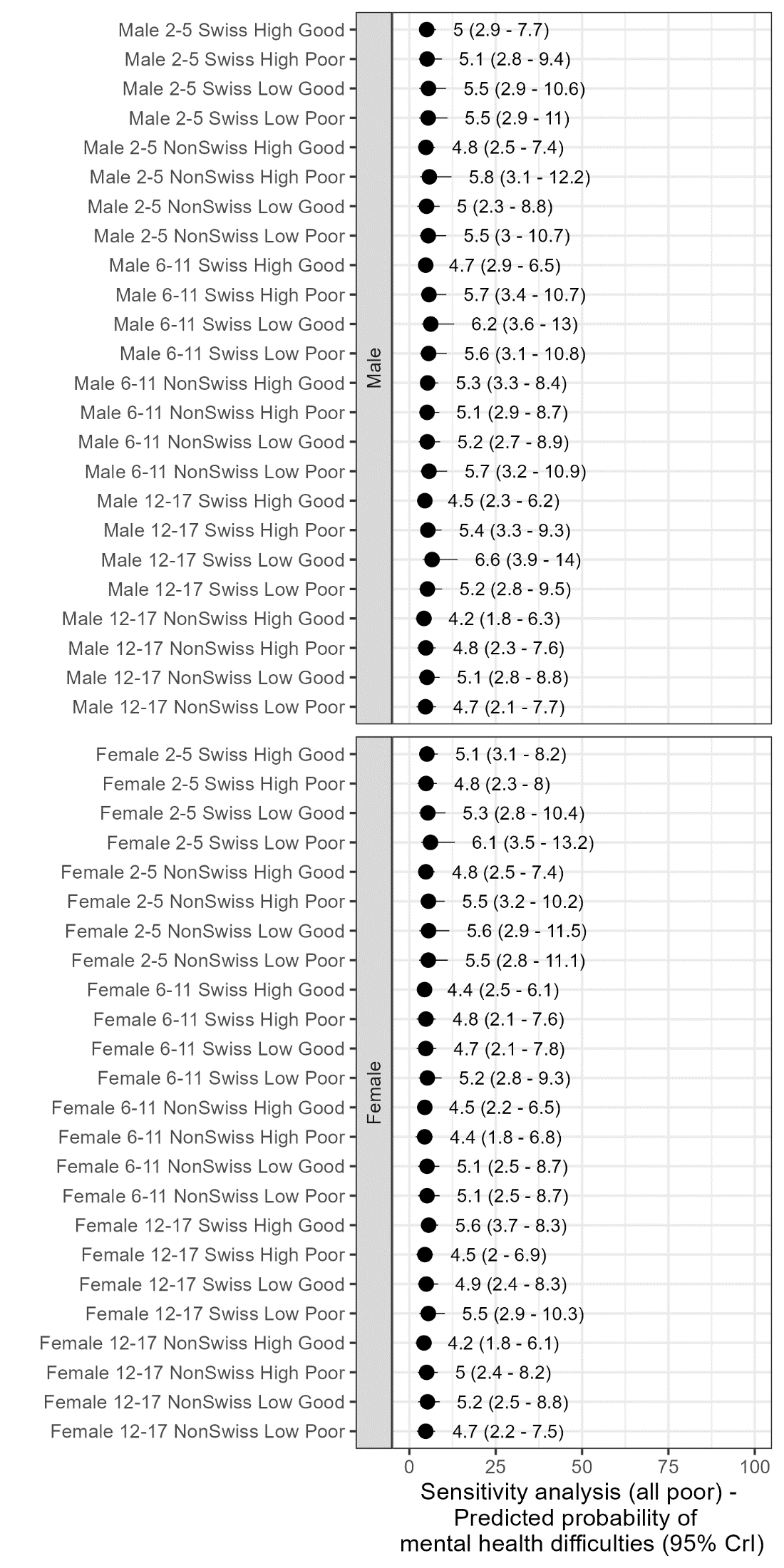

Supplement: Multimedia component 1 [file mmc1.docx]
